# Editorial Notes

**Published:** 1883-08

**Authors:** 


### EDITORIAL NOTES.

Dr. L. S. McMurtrey has retired from editorial connection with the *Louisville Medical News*, and is succeeded by Dr. H. A. Cottell, formerly editorial associate of Profs. Cowling and Holland. We must express our regret at the retirement of Dr. McMurtrey; a forcible and attractive writer, his talents have always been directed to the promotion of the best interests of the profession.

We call the attention of our readers to the advertisement on another page, headed "Food Preservatives." That it is of the first importance to prevent septic or putrefactive changes in animal and vegetable matter intended for food, will at once be admitted. Some incredulity is to be expected as to the ability to accomplish this desirable end, without either injuring the taste or healthfulness of food so treated. The Humiston Food Preserving Company, however, claim to have completely succeeded, and their claim is supported by the testimony of Prof. Samuel Johnson of Yale College, one of the most accomplished chemists in the country, as well as by many medical men.

The first number of the *Journal of the American Medical Association*, issued under date of July 14th, was duly received, and the second number has just come to hand. It is a double column, quarto page journal, very similar in appearance to the *British Medical Journal*. As yet, however, the similarity extends only to the printer's work. The editorials, original articles and selections are as good as the average medical journal presents to its readers. It is unfair, however, to expect too much, or to form an opinion of what the journal will be from the first few numbers. We hope and expect that it will in time take a leading position among the medical journals of the world.

NOTHING is too absurd for some one to assert, and nothing is too silly for many to believe, but it is painful to see medical men allowing their imaginations to carry them into the realms of the "Lunatic, the lover and the poet," as instanced in the following *official* report of the post-mortem examiners as to the cause of the death of Capt. Webb, who lost his life in the attempt to swim through the rapids below Niagara Falls:

"As the result of our examination, I am led to the conclusion, and in this I am seconded by my colleagues, that death was caused, not by asphyxia or drowning, or by any local injury by the body coming in contact with any hard substance, but by the shock from the reactionary force of the water in the whirlpool rapids coming in contact with the submerged body with such force as to instantly destroy the respiratory power, and, in fact, all vital action by direct pressure and force of contact—a shock of sufficient intensity to paralyze the nerve centers, partially desiccate the muscular tissues, and forestall any probable sequel of death by drowning."

